# Rapid cortical dynamics associated with auditory spatial attention gradients

**DOI:** 10.3389/fnins.2015.00179

**Published:** 2015-06-02

**Authors:** Jeffrey R. Mock, Michael J. Seay, Danielle R. Charney, John L. Holmes, Edward J. Golob

**Affiliations:** ^1^Department of Psychology, Tulane UniversityNew Orleans, LA, USA; ^2^Program in Neuroscience, Tulane UniversityNew Orleans, LA, USA; ^3^Program in Aging, Tulane UniversityNew Orleans, LA, USA

**Keywords:** attention, spatial hearing, EEG, laterality, independent component analysis

## Abstract

Behavioral and EEG studies suggest spatial attention is allocated as a gradient in which processing benefits decrease away from an attended location. Yet the spatiotemporal dynamics of cortical processes that contribute to attentional gradients are unclear. We measured EEG while participants (*n* = 35) performed an auditory spatial attention task that required a button press to sounds at one target location on either the left or right. Distractor sounds were randomly presented at four non-target locations evenly spaced up to 180° from the target location. Attentional gradients were quantified by regressing ERP amplitudes elicited by distractors against their spatial location relative to the target. Independent component analysis was applied to each subject's scalp channel data, allowing isolation of distinct cortical sources. Results from scalp ERPs showed a tri-phasic response with gradient slope peaks at ~300 ms (frontal, positive), ~430 ms (posterior, negative), and a plateau starting at ~550 ms (frontal, positive). Corresponding to the first slope peak, a positive gradient was found within a central component when attending to both target locations and for two lateral frontal components when contralateral to the target location. Similarly, a central posterior component had a negative gradient that corresponded to the second slope peak regardless of target location. A right posterior component had both an ipsilateral followed by a contralateral gradient. Lateral posterior clusters also had decreases in α and β oscillatory power with a negative slope and contralateral tuning. Only the left posterior component (120–200 ms) corresponded to absolute sound location. The findings indicate a rapid, temporally-organized sequence of gradients thought to reflect interplay between frontal and parietal regions. We conclude these gradients support a target-based saliency map exhibiting aspects of both right-hemisphere dominance and opponent process models.

## Introduction

Audition is distinguished from the other major senses by the ability to panoramically monitor the environment for things happening at a distance, behind obstructions, and out of sight. These ecological considerations suggest that the auditory system is particularly useful for shifting spatial attention to events that are important for survival and reproduction. The sound of a snapping twig, for example, can disclose the location of an approaching predator in time to prepare a fight or flight response.

The properties of spatial attention have been intensively studied in the visual modality. It is well-established that attention can be expressed as a spatial gradient relative to an attended location (Wachtel, 1967; Downing and Pinker, 1985; Rizzolatti et al., 1987; Mangun and Hillyard, 1988; Handy et al., 1996; Cave and Bichot, 1999; Intriligator and Cavanagh, 2001). Similar observations suggestive of an attention gradient are found in patients with hemineglect (Kinsbourne, 1993). The term “attention gradient” is a compact way to convey the idea that attentional benefits can progressively decrease with greater distances from the current focus of spatial attention. Presumably the need for an attention gradient is a byproduct of having capacity limitations, although selectivity could also reflect limitations in behaviors that are possible at one time (Allport, 1989).

Previous behavioral studies of auditory spatial attention have shown that both endogenous (top-down) and exogenous (bottom-up) cuing at a given location can facilitate reaction times to subsequent targets at the cued location relative to another location (Spence and Driver, 1994), several locations (Rhodes, 1987; Mondor and Zatorre, 1995; Rorden and Driver, 2001), and between different sensory modalities (Spence et al., 1998, 2000). Some of the strongest evidence for auditory attentional gradients was reported by Mondor and Zatorre (1995) and Rorden and Driver (2001). Both groups used a cue to orient auditory attention in space and then presented a target shortly afterwards, and both found that reaction times increased monotonically with increases in the distance between the cue and target locations.

Auditory studies of attention gradients using EEG methods find attentional effects over a restricted spatial range during rapid stimulus presentation. During passive listening infrequent changes in location elicit the mismatch negativity potential (latency ~100–150 ms), which has progressive increases in amplitude up to ~30–35° (Arnott and Alain, 2002b; Deouell et al., 2006). During selective attention tasks where stimuli are presented rapidly from closely spaced locations, which imposes a high perceptual load, ERP measures also reflect an attention gradient (Teder-Salejarvi and Hillyard, 1999; Teder-Salejarvi et al., 1999; Arnott and Alain, 2002a,b). The main ERP measure in these studies was a biphasic potential from ~100–500 ms that is more negative for attended relative to ignored distractors (Hillyard et al., 1973).

Neuroimaging studies implicate a network centered on bilateral dorsal frontal and parietal regions in top-down attention control and a more ventral, right hemisphere set of frontoparietal regions in bottom-up control (Corbetta and Shulman, 2002). Imaging studies show activations in prefrontal and parietal regions after cueing attention shifts are similar for auditory and visual tasks (Wu et al., 2007). However, there may also be substantial modality differences, such as predominantly right hemisphere activations (Zatorre et al., 1999) or activations that indicate contralateral mapping between hemisphere and attended location that are evident only at a fine-grained level using machine learning analysis (Kong et al., 2014). Shifting attention is particularly associated with parietal activations, and has been studied in visual, auditory, and shifts between visual and auditory modalities (Yantis et al., 2002; Shomstein and Yantis, 2004, 2006).

We recently built on this literature by presenting sounds under more ecologically typical conditions, with slower presentation rates and greater separation between sound locations (Golob and Holmes, 2011). We employed a spatial variant of the classic oddball task (Sutton et al., 1965; Polich, 2007), where participants attended to a target location on either the left (−90°) or right (+90°) side of the head in the horizontal plane, and pressed a button when white noise was presented at the target location (Golob and Holmes, 2011). Stimuli were presented at 5 equally likely locations, including the target and 4 non-target distractors spaced in 45° increments. When ERPs to distractors were examined as a function of their distance (angle) from the target location we found that several potentials between ~200–800 ms (P200, P3a, slow waves) had progressive increases in amplitude as the distractor was placed farther away from the target (up to 180°). These progressive increments in amplitude were interpreted either as indexing an increase in the strength of bottom-up orienting during distractor processing or as indicating a decrement in the extent to which distractors recruited top-down attentional resources. In both cases results were consistent with interpretation of a neuroelectric correlate of spatial attention gradients. However, additional analyses to further characterize the timing and cortical sources of gradients were not performed.

There were two main purposes of this study. We first examined scalp data to identify rapid shifts in spatial attention gradients. This was accomplished using a novel approach in which a linear regression model defined the slope of ERP amplitudes to distractor sounds across four different distances in relation to an attended location. The slope measure was used because it provides a simple expression of linear differences, some of which are not evident having an ERP peak. The slope across spatial locations were calculated every 2 ms after distractor sound onset in order to identify the timing of ERP gradients, which are indicated by the presence of significant positive or negative slopes relative to 0. We report new findings that suggest a tri-phasic attentional gradient response, with distinct topographies that alternate between frontal and parietal regions. Second, we performed independent component analysis (ICA) to decompose scalp EEG data into cortical sources with distinct event-related amplitude and oscillatory responses. Finally we discuss our results in relation to competing theories of spatial attention.

## Materials and methods

### Participants

Thirty-five healthy university students (15 males, 20 females; age = 21 ± 1.0 years; education = 14.3 ± 0.3 years; 34 right-handed, 1 left-handed) participated in the study. Sixteen of the 35 participants were included in a previous study (Golob and Holmes, 2011). Pure tone thresholds were tested from 500 to 8000 Hz using an audiometer (Maico, Eden Prairie, MN) to ensure that hearing thresholds were <25 dB (0.5–4 kHz) and differences between ears were <10 dB. Each participant gave written informed consent, and the experiments were performed in accordance with a protocol approved by the Tulane University Institutional Review Board that was consistent with the Declaration of Helsinki.

### Design and rationale

A cardinal feature of attention is selectivity of information processing. We used a task where a region of space is selected for preferential processing by asking participants to attend either to a left or right target location. The choice of far left and right locations permitted a wide range of spatial locations to be tested for defining attentional gradients. In separate blocks participants attended to a location on either the left (−90°) or right (+90°) side of the head in the horizontal plane, and pressed a button when broadband noise was presented at the target location. Stimuli were presented at 5 locations, including the target and 4 non-target locations, spaced in 45° increments. The EEG responses to non-target distractors that show a gradient would potentially index attention processes such as shifting attention to the distractor's location and back to the target location.

### Stimuli

Five virtual white noise burst sounds (0.1–10 kHz, 200 ms duration, 5 ms rise/fall times, ~60 dB nHL) were created to correspond to five locations in the frontal azimuth plane (left to right: −90°, −45°, 0°, +45°, +90°). The spatialized sounds were created by applying appropriate interaural time and level differences as well as head related transfer functions for each spatial location, which were based on a KEMAR model supplied by Tucker-Davis Technologies (Gainesville, Florida, USA, System II and the University of Wisconsin). The same initial white noise sample was processed to generate stimuli for each location. The stimuli provided the same basic directional cues to the auditory system as those used to define sound location under natural conditions (Yost and Gourevitch, 1987). Stimuli were presented with insert earphones (Compumedics-Neuroscan, Charlotte, NC) with a passband extending above 10 kHz. Insert earphones were used rather than free-field speakers in order to limit the influence of visual indicators of sound sources and avoid changes in the relationship between sound source location and the ears due to head movements.

### Experimental paradigm

Before the experiment began each participant underwent testing to determine if they could accurately perceive the location of each sound. Stimuli corresponding to each of the five sound locations was repeatedly presented (stimulus onset asynchrony = 750 ms), and participants marked the perceived sound location on a sheet of paper with a semicircle overlaid onto perpendicular lines representing the midline and interaural axes (Blauert, 1997). After familiarization all participants could accurately report each perceived sound location, with some variability in reporting the ±45° locations. Increased variability in localization of the ±45° sounds was expected because listeners make larger errors in sound localization between 40° and 60° in the azimuthal plane (Makous and Middlebrooks, 1990).

**Figure 1:**
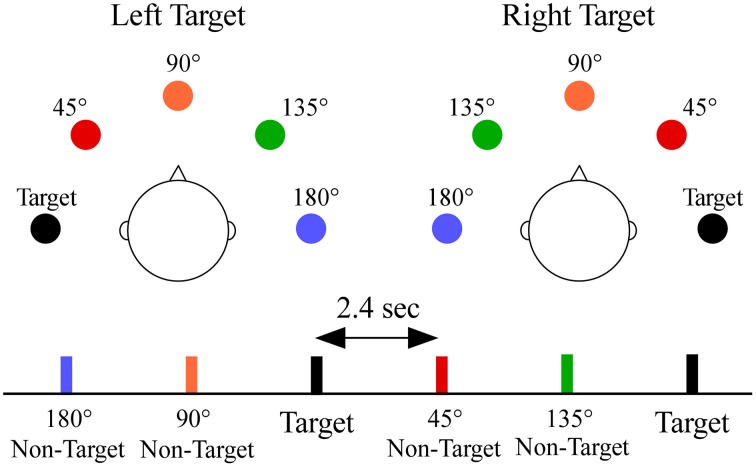
**Overhead schematic of the virtual sound locations**. The sound locations are shown relative to the listener's head for each target location, and are color-coded to a segment of a stimulus sequence. Subjects were required to respond by button press only to sounds appearing at one target location [−90° **(left)** or +90° **(right)**] within a block. Non-targets were classified by their angular distance from the target location (termed angular disparity). Sounds at each location were equally probable and randomly presented with a constant stimulus onset asynchrony of 2.4 s.

A schematic showing stimulus configuration and a sample sequence is shown in Figure 1. During testing each participant held a response pad while listening to sequences in which white noise was presented semi-randomly from each of the five sound locations. Each participant was told to face forward, look straight ahead, and respond as quickly as possible while ensuring accurate responses to a designated target location (−90° or +90°, in separate blocks, order counterbalanced across participants) by pressing a button with the thumb of their dominant hand. Sixteen of the 35 participants also completed blocks of trials in which the target appeared at 0°; which was not analyzed for this paper. Each location had a 0.20 probability (target *p* = 0.20, non-target *p* = 0.80, 0.20 probability/non-target) and was randomly presented within each block (stimulus onset asynchrony = 2.4 s). There were 150 stimuli per block. Each target location had two blocks for a total of four blocks. Behavioral measures to targets included median reaction time, hit rate (percentage of responses to target) and false alarm rate (percentage of responses to non-targets).

### EEG recordings

The experiment was conducted in a sound attenuating, electrically shielded booth (IAC Acoustics, Bronx, New York). An electrode cap containing 64 Ag/AgCl electrodes positioned in accordance with the 10/20 system was placed on the scalp of each participant with the reference electrode between Cz and CPz (impedances ≤ 10 kΩ). Four electrodes were used to monitor eye movements, one above and one below the left eye and one lateral to each eye. The EEG and electro-oculogram were continuously digitized at 500 Hz (DC, 100-Hz bandpass) with a 64-channel EEG system (Compumedics-Neuroscan, Charlotte, NC, USA) and stored for off-line analysis.

### EEG analysis: scalp channels

**Figure 2:**
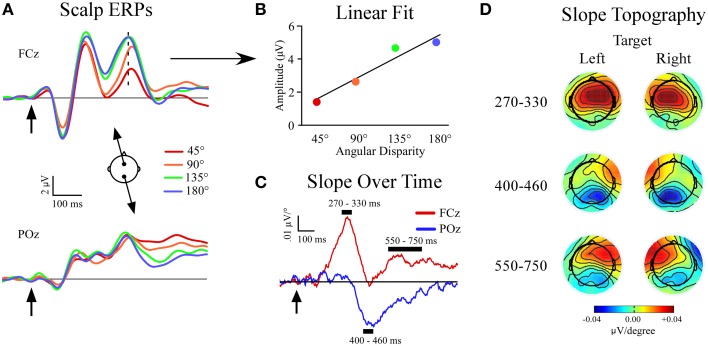
**Slope of event-related potentials (ERPs) across non-target locations. (A)** Event-related potentials to non-targets recorded at midline sites FCz and POz. Separate non-target ERPs are plotted by their angular disparity from the target location. **(B)** Demonstration of the least-squares linear fit procedure for *t* = 320 ms at FCz (indicated by dotted line in ERP). **(C)** Slope values plotted for each time-point at FCz and POz. **(D)** Topography of slope values across all channels within specified time windows and plotted separately for left (−90°) and right (+90°) target conditions. Arrows indicate stimulus onset.

For scalp channel analysis electrodes were referenced in a linked mastoid configuration and corrected for DC drift and eye blink artifacts (Gratton et al., 1983). The data were epoched into 1200 ms segments (−200 to 1000 ms relative to stimulus onset) and visually inspected for artifacts such as large muscular potentials or electrode movement. Individual sweeps were then sorted and averaged based on sound location (4 non-targets and 1 target/condition), and baselined from −100 to 0 ms relative to stimulus onset. All sweeps that included incorrect responses (missed targets and false alarms to non-targets) were excluded from the ERP averages. Our previous study showed linear amplitude increases in certain non-target ERP peaks (P3a, slow wave) as the angular distance between the target and non-target location widened (Golob and Holmes, 2011). To further investigate this phenomenon we fit a linear function to the voltage at each sample point and electrode site across the four non-target locations relative to the target location (45°, 90°, 135°, and 180° from target location; Figures 2A,B). In effect, the slope measure provides a condensed expression of stimulus-evoked EEG responses as a function of distance from the target location. For each linear fit the slope and goodness-of-fit (*r*^2^) were recorded. The slope measure produced three peaks, starting with a positive peak at frontal sites (270–330 ms). The second peak had a negative slope at posterior sites (400–460 ms), while the final peak had a positive slope that was maximal at frontal sites contralateral to the target location (550–750 ms; Figures 2C,D).

### EEG analysis: independent component analysis (ICA)

For ICA, continuous EEG data were first imported into EEGLAB 12.0 (Delorme and Makeig, 2004), which is an open-source software toolbox (Swartz Center for Computational Neuroscience, La Jolla, CA; http://sccn.ucsd.edu/eeglab/) that was run in Matlab version 8.0 (The Mathworks, Inc., Natick, MA). A 38-channel subset of electrodes was selected for further analysis. The choice of 38 sites was based on having an adequate sampling of spatial scalp sites while also limiting the number of channels and computation time. The 38 electrode sites were equally distributed within frontal to posterior (FP to O) and medial to lateral (z to 7/8) sites based on the 10–20 system. The EEG was high-pass filtered at 1 Hz, re-sampled at 250 Hz, and re-referenced to the average of all 38 scalp channels. The resulting continuous data were segmented into epochs from 1200 ms before to 1200 ms after stimulus onset. A longer epoch was used for the ICA analysis because ICA solution strength (i.e., amount of mutual information reduction between components) increases when applied to a larger number of time points and because accurate time-frequency analysis at lower frequencies (<5 Hz) requires a longer time window. The data were visually inspected for the presence of outlying data and non-stereotyped artifacts. Channels containing non-stereotyped artifacts throughout the recording (e.g., line noise) and epochs containing other non-stereotyped artifacts (muscular potentials, scalp-electrode connectivity or movement) were removed from the data.

After the data rejection procedure there was a mean of 35 ± 1 channels and 332 ± 11 epochs per participant. We then performed extended infomax ICA on the data (Bell and Sejnowski, 1995). Independent component analysis finds an unmixing square matrix with rows and columns equal to the number of input channels which, when matrix-multiplied with the raw data, provides maximally temporally independent activations. Each independent component (IC) activation has a fixed topographic projection map to scalp channels which is given by the inverse of the unmixing matrix. Independent components for each participant were accepted for further analysis based on scalp map topography (smooth regions of positive and negative polarity that are well-distributed across channels), mean log spectrum (1/f-like curve with typical EEG spectral peaks, e.g., θ, α, β frequency bands), and consistent trial-to-trial activations as evidenced by time-locked peaks in epoch averages (ERPs) and corresponding peaks in trial activations. Independent components having characteristics indicative of stereotyped artifacts (eyeblinks, eye movements, electrocardiogram, and muscular potentials) were removed. A total of 512 ICs (mean = 15 ± 1 per participant) were selected for further analysis.

The neural source of the selected ICs was modeled using DIPFIT2 using functions from the FIELDTRIP toolbox [(Oostenveld et al., 2011); Donders Institute for Brain, Cognition and Behavior; http://fieldtrip.fcdonders.nl/]. DIPFIT2 uses scalp topographic maps as an input, and calculates the location and orientation of a single equivalent current dipole using a three-shell boundary element model. A standard boundary element head model was used for all participants and was composed of three 3-D surfaces (skin, skull, cortex) extracted from the Montreal Neurological Institute (MNI) canonical template brain. Scalp channel locations were co-registered with locations in the model space by aligning them with their standard locations in the 10–20 system relative to the MNI template.

Time-frequency analysis of the IC activations, known as the event-related spectral perturbation (ERSP), was also calculated. The ERSP visualizes mean event-related changes in spectral power over time in a broad range of frequency bins. In doing so the ERSP generalizes classic event-related desynchronization and synchronization measures (Pfurtscheller and Aranibar, 1977). Time-frequency analysis of single-trial IC activations was performed by convolving the data with a Morlet wavelet that used 3 cycles at the lowest frequency (2.5 Hz) and a linearly increasing number of cycles up to 30 at the highest frequency (50 Hz). The result was scaled to decibels (dB), and the values in the post-stimulus period were normalized for each frequency by subtracting the mean value in the baseline period. The ERSP was imaged by plotting the normalized power values as a color within a “heat map” in a 2-D time-frequency plot.

A clustering procedure was used to determine which ICs from different participants represented similar functionally distinct EEG processes. We first pre-defined and computed four measures for each IC: scalp map, dipole location, ERP, and ERSP. For each measure a data space was constructed in which measures could be compared across all ICs. Principal component analysis was applied to separate the IC data along a pre-defined number of orthogonal dimensions based on both spatial (scalp map and dipole location) and non-spatial (ERP and ERSP) features. The resulting principal component templates were concatenated and principal component analysis was then applied to reduce the total number of dimensions by half. This resulted in a set of data in which each component possessed a value in a 15-dimensional data space. We then applied a *k*-means clustering algorithm to this data space which separates components into k clusters and observed the results for *k* = 8 to *k* = 15. During the clustering process, we also removed components whose centroids were >3 SDs from the centroid of any cluster metric space. Based on the consistency of clustering solutions for increasing values of *k*, we identified six clusters of interest that had both a high proportion of participant contribution and whose activities contained significant effects. If a subject contributed more than one component to a given cluster two of the authors (JM and MS) independently chose one component per participant within each of the six clusters of interest based on assessment of the measures used for clustering. The two independent sets of IC placements agreed on 459 out of the 512 total ICs (89.6%). For the remaining 53 disagreements, the two authors compared the relevant ICs and came to an agreement for the final clustering.

### Statistical analysis

Unless otherwise specified, we used repeated measures analysis of variance (ANOVA) tests (significance = *p* < 0.05). Behavioral measures included hit rate, reaction time, and false alarm rate. Hit rate and reaction time were analyzed by target location (−90°, +90°), and false alarm rate included an additional factor of angular disparity (45°, 90°, 135°, 180°). Angular disparity is the angular difference between the locations of a distractor and the attended target. For example, when attending to the left (−90°) the distractors from left to right (−45°, 0°, +45°, +90°) have angular disparities of 45°, 90°, 135°, and 180°, respectively.

Factors for the slope analysis included target location (−90°, +90°) and electrode site (for midline analysis: frontal, FCz, and posterior, POz; for laterality analysis: left, FC5, PO5, and right, FC6, PO6). Although differences across electrode sites are often captured visually in topographic plots, we included the factor of electrode site to statistically demonstrate hemispheric and anterior/posterior differences. Preliminary analyses of nearby posterior sites (PO7/PO8) yielded the same results. The midline and lateral electrodes were analyzed separately for each mean slope time window (270–330 ms, 400–460 ms, 550–750 ms). Previous work found small decreases in P3a latency for non-targets farther from the target location (Golob and Holmes, 2011). Potential latency differences would not affect the analysis here because the mean slope time windows are much larger than any small latency effects. When appropriate, frontal and posterior electrode sites were analyzed separately. To determine whether a significant linear slope was present single sample *t*-tests were tested relative to zero slope.

Clusters composed of ICs included measurements of ERPs and ERSPs that were initially assessed using *t*-tests in sliding windows following stimulus onset. Based on these results significant time and frequency ranges of interest were identified in each cluster, and mean measures within these regions were quantified for each participant. Repeated measure ANOVA was used to analyze the IC ERP activations and ERSP power values within each cluster separately, using factors of target location (−90°, +90°) and angular disparity (45°, 90°, 135°, 180°). Based on previous observations (Golob and Holmes, 2011), we hypothesized a linear relationship across angular locations and therefore report results of linear contrasts within the ANOVA tests. One analysis used absolute locations relative to the head, which will be specified below.

## Results

### Behavioral results

The mean reaction time to targets was 573 ms ± 17 ms with no difference between target locations (−90° = 579 ± 19 ms; +90° = 563 ± 18 ms). However, target hit rate was somewhat greater when attending to the −90° vs. +90° target location [*t*_(34)_ = 2.2, *p* = 0.03; −90° = 80% ± 2%, +90° = 76% ± 3%]. False alarms to distractors were comparable for the −90° vs. +90° target location (−90° = 2.5% ± 0.4%, +90° = 2.5% ± 0.4%). There was a main effect of angular disparity [*F*_(3, 32)_ = 11.8, *p* < 0.001] because most false alarms occurred at the 45° location nearest to the target (nearest 45° = 9.5% ± 1.6%, other locations = 0.2% ± 0.1%).

The EEG findings will be presented in two sections. First we present analyses that used linear functions to determine the slope of EEG voltage at each time-point after stimulus onset (−100–900 ms, every 2 ms) across the four distractor locations. The second section will report ICA results, with a focus on components that contribute to the peaks of the slopes identified in the first section.

### Slopes of EEG voltage across distractor locations

The grand average ERPs are shown in Figure 2A, and a schematic of the slope fitting procedure is shown in Figure 2B. Results showed three slope peaks (Figures 2B,C) at different time points after stimulus onset (270–330 ms, 400–460 ms, 550–750 ms).

#### 270–330 ms window

There was a main effect of electrode site [*F*_(1, 34)_ = 127.3, *p* < 0.001] due to the development of a positive frontal slope (FCz: slope = 0.041 ± 0.005 μV/°). A single-sample *t*-test showed that the slope differed from zero at FCz (*p* < 0.001) with mean *r*^2^ = 0.61 ± 0.03. The slope did not differ from zero at POz (*p* = 0.6).

At the lateral electrodes, a main effect of electrode site was found [*F*_(1, 34)_ = 94.8, *p* < 0.001] but was superseded by a target location x electrode site x hemisphere interaction [*F*_(1, 34)_ = 33.7, *p* < 0.001]. Both frontal lateral electrodes showed a positive slope that differed from zero. However, the positive frontal slope was larger in the contralateral hemisphere of the target location (*p* = 0.004). No posterior lateral electrodes showed a slope that differed from zero (Figure 2D).

#### 400–460 ms window

There was a main effect of electrode site [*F*_(1, 34)_ = 33.6, *p* < 0.001]. A negative slope developed posteriorly (POz: slope = −0.029 ± 0.003 μV/°). A single-sample *t*-test showed the slope differed from zero at POz (*p* < 0.001) and had a mean *r*^2^ = 0.44 ± 0.04. There was no frontal midline slope at FCz (*p* = 0.8).

The lateral electrodes showed a main effect of electrode site [*F*_(1, 34)_ = 111.2, *p* < 0.001], but was superseded by a target location x electrode site x hemisphere interaction [*F*_(1, 34)_ = 17.8, *p* < 0.001]. The frontal lateral electrodes showed a positive slope that differed from zero only in the contralateral hemisphere of the target location (contralateral: *p* = 0.0001, ipsilateral: *p* = 0.1). Also, both posterior lateral electrodes showed a negative slope that differed from zero (contralateral: *p* < 0.0001, ipsilateral: *p* < 0.0001). However, the negative posterior slope was larger in the contralateral vs. ipsilateral hemisphere of the target location (*p* < 0.0001, Figure 2D).

#### 550–750 ms window

A main effect of electrode site was found [*F*_(1, 34)_ = 30.2, *p* < 0.001]. A positive slope developed frontally (FCz: slope = 0.015 ± 0.004 μV/°), and was different from zero (*p* = 0.0007). There was also a posterior negative slope (POz: slope = −0.015 ± 0.005 μV/°) that differed from zero (*p* = 0.0003) and may reflect residual activity of the second slope peak. The *r*^2^-value was similar across target location and electrode site (*r*^2^ = 0.40 ± 0.02). Paired comparisons showed that the *r*^2^-value in the 550–750 ms window was less than at 270–330 ms at FCz (*p* < 0.001), and trended towards a difference at 400–460 ms at POz (*p* = 0.059). The reduced linear fit at the last latency window (550–750 ms) quantifies the impression that the function of voltage vs. stimulus location starts to become nonlinear at longer latencies, possibly reflecting a more focal attention gradient (Golob and Holmes, 2011).

At lateral electrodes a target location x electrode site x hemisphere interaction was found [*F*_(1, 34)_ = 14.1, *p* = 0.001]. The frontal electrodes showed a slope that differed from zero only in the contralateral hemisphere of the target location (contralateral: *p* < 0.0001, ipsilateral: *p* = 0.3) while both lateral posterior electrode showed a negative slope that differed from zero irrespective of the target location (contralateral: *p* < 0.0001, ipsilateral: *p* < 0.0001).

### Independent component analysis: overview

**Figure 3:**
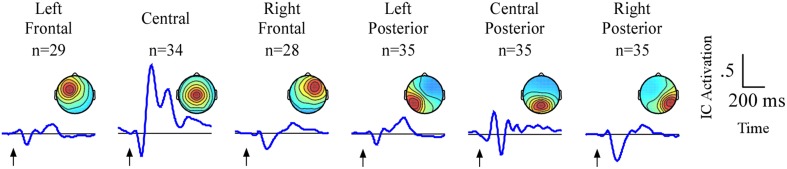
**Result of independent component clustering procedure**. Six clusters were found. For each cluster, mean scalp map and grand average ERPs of independent component activations are plotted. Arrows indicate stimulus onset.

The analyses will focus on six clusters of ICs in which attention-related effects were identified. An average of 33/35 participants contributed an IC to each cluster (Figure 3). Two frontal (left frontal and right frontal) and one central cluster exhibited spectral peaks in the theta (5–8 Hz) band while three posterior clusters (left posterior, central posterior and right posterior) exhibited spectral peaks in the alpha (9–12 Hz) band. Based on the ERP and ERSP data from each cluster, we identified several time and frequency regions containing attention-related differences. A summary of the independent component results is presented in Table 1.

**Table 1:** **Summary of independent component slope results**.

**Measure**	**Target location**	**Left frontal**	**Central anterior**	**Right frontal**	**Left posterior**	**Central posterior**	**Right posterior**
ERP	−90°		+	+	−	−	−
			200–360 ms	200–360 ms	120–200 ms	360–500 ms	440–600 ms
	+90°	+	+		+	−	+
		200–360 ms	200–360 ms		120–200 ms	360–500 ms	320–440 ms
ERSP	−90°					− β	− αβ
						250–550 ms	250–550 ms
	+90°				− αβ	− αβ	
					250–550 ms	250–550 ms	

*Summary of independent component slope results by cluster, target location, and measurement. Significant results are represented in the table by the presence of a slope (quantified by a significant linear contrast). Slope direction is indicated by a sign (+ or -) along with the time window in which the data was analyzed. For ERSP slope findings, the frequency band in which the slope was found is also indicated (α, β)*.

### Independent component analysis: event-related potential (ERP) activation

#### Central cluster

**Figure 4:**
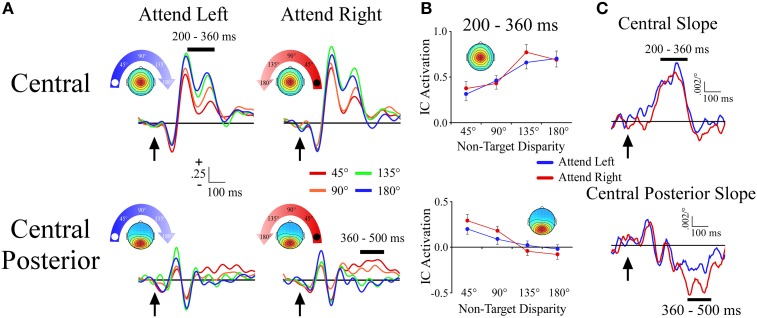
**ERPs from midline ICA clusters. (A)** Event-related potentials for the Central and Central Posterior clusters shown separately for −90° (left) and +90° (right) target locations. For each cluster and target location, the ERPs are plotted according to their angular disparity from the target location. **(B)** Mean independent component activations across angular disparity within time windows, which are indicated by black bars above the ERPs. **(C)** Slope of independent component activations across non-target disparity as a function of time. Arrows indicate stimulus onset.

The central cluster exhibited a positive ERP slope between non-target locations from 200 to 360 ms, and corresponded well to the first slope peak described above (Figures 4A,B). The positive slope was represented by an effect of angular disparity [*F*_(1, 33)_ = 31.9, *p* < 0.001] in which mean IC ERP activations of non-target sounds increased linearly with greater angular disparity from the target location. This effect was independent of target location as there was no main effect or interaction that involved target location.

#### Central posterior cluster

The central posterior cluster tapped into activity at the time of the second slope peak described above, with a negative ERP slope between non-target locations from 360 to 500 ms (Figures 4A,B). The negative slope was represented by an effect of angular disparity [*F*_(1, 34)_ = 36.2, *p* < 0.001], where IC ERP activations decreased linearly with increasing angular disparity from the target location. This effect was comparable among target locations as there was no main effect or interaction that involved target location. We note that Figure 4B also shows a small positive slope at ~140 ms. For present purposes we will focus on the largest slope peaks, but future work may examine this and other smaller slope peaks that may be present at shorter latencies.

#### Left and right frontal clusters

**Figure 5:**
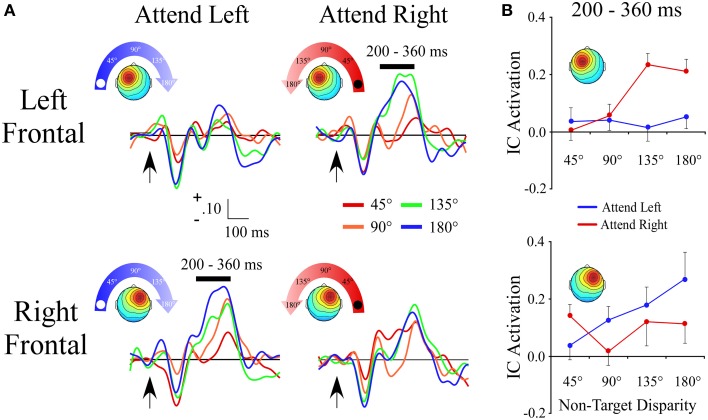
**ERPs from lateral frontal ICA clusters. (A)** Event-related potentials for the Left Frontal and Right Frontal clusters shown separately for −90° (left) and +90° (right) target locations. For each cluster and target location, the ERPs are plotted relative to the angular disparity from the target location. **(B)** Mean independent component activations across angular disparity within time windows indicated by black bars above ERPs. Arrows indicate stimulus onset.

The lateral frontal clusters exhibited a positive ERP slope between non-target locations in the same time range (200–360 ms) as the central cluster. However, as seen by a target location x angular disparity interaction within the left frontal [*F*_(1, 28)_ = 16.8; *p* < 0.001] and right frontal clusters [*F*_(1, 27)_ = 7.5; *p* = 0.01], the IC ERP activations had a linear slope only when the target location was contralateral to the hemisphere with the maximum of the cluster's topographic map (Figures 5A,B).

#### Left and right posterior clusters

**Figure 6:**
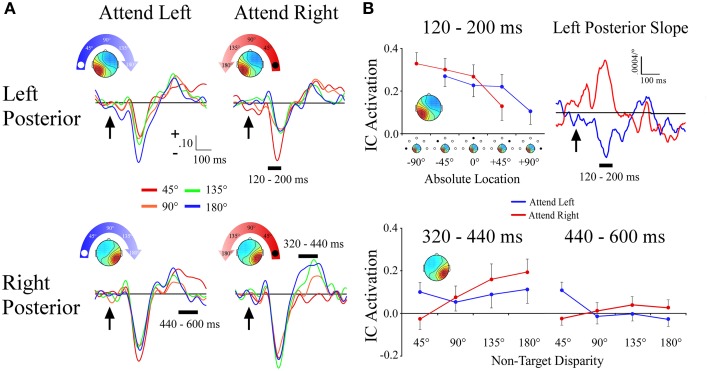
**ERPs from lateral posterior ICA clusters. (A)** Event-related potentials for the Left Posterior and Right Posterior clusters shown separately for −90° (left) and +90° (right) target locations. For each cluster and target location, separate ERPs are plotted by their angular disparity from the target location. **(B)** Mean independent component activations across angular disparity within time windows indicated by black bars above ERPs. For the Left Posterior cluster, the slope of independent component activations across non-target disparity is plotted over time. Arrows indicate stimulus onset.

Unlike the lateral frontal clusters, the lateral left and right posterior clusters differed in both the time course of the effects and in terms of coding for absolute vs. attention based reference frames. Within a 120–200 ms time window, the left posterior cluster showed a target location x angular disparity interaction [*F*_(1, 34)_ = 20.5; *p* < 0.001] that reflected absolute space. Activation within the left posterior cluster became more negative the farther right along the frontal azimuth plane the distractor sound was presented, irrespective of the target location. This effect was further supported by observation of nearly mirror-image slopes during 120–200 ms for −90° and +90° target locations (Figures 6A,B). No other effects were observed within the left posterior cluster.

The right posterior cluster had a target location x angular disparity interaction [*F*_(1, 34)_ = 7.0; *p* = 0.01], within a 320–440 ms time window. This interaction was due to the IC ERP activations within the right posterior cluster showing a positive linear slope only when attending to the ipsilateral (+90°) target location (Figures 6A,B). Within a later 440–600 ms time window the right posterior cluster also had a target location x angular disparity interaction [*F*_(1, 34)_ = 9.2; *p* = 0.005], due to a negative linear slope when attending to the contralateral (−90°) location.

### Independent component analysis: event-related spectral perturbations (ERSPs)

#### Central posterior cluster

##### Alpha band (8–12 Hz)

**Figure 7:**
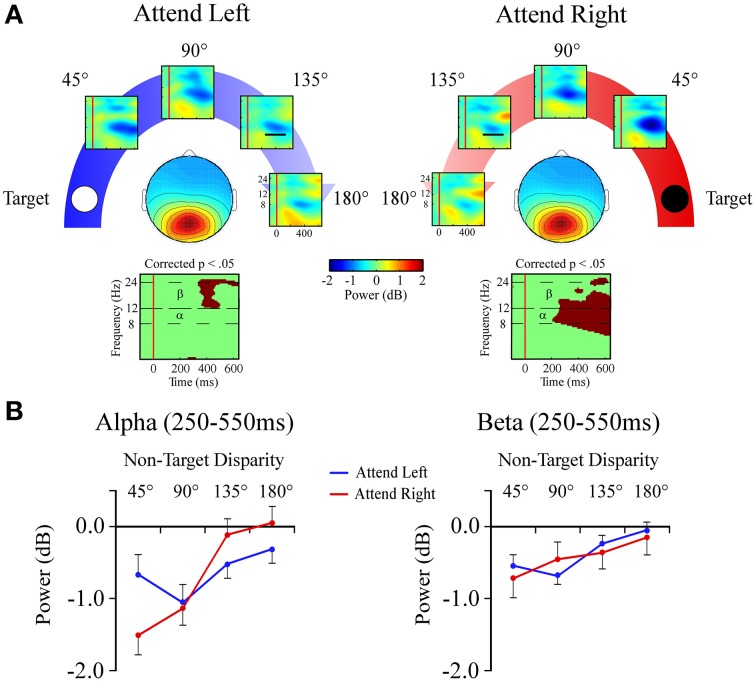
**Event-related spectral perturbations (ERSPs) from the Central Posterior ICA cluster. (A)** Central Posterior cluster ERSP plots shown separately for −90° (left) and +90° (right) target locations. Plots below each scalp map highlight time-frequency points for which *p* < 0.05 in a *t*-test across angular disparity after correcting for False Discovery Rate. **(B)** Mean ERSP power across angular disparity in separate plots for alpha (α, 8–12 Hz) and beta (β, 12–24 Hz) bands within a 250–550 ms time window (indicated by black bar in the 135° ERSP plot).

In the alpha band there was a target location x angular disparity interaction [*F*_(1, 34)_ = 5.9; *p* = 0.001; Figures 7A,B]. When attending to the +90° target location, a linear decrease in alpha power, indicating a desynchronization, was measured as the distractor sound was presented farther from the target location.

##### Beta band (12–24 Hz)

For beta band measures there was a main effect of angular disparity [*F*_(1, 34)_ = 31.3; *p* < 0.001; Figures 7A,B]. This showed that irrespective of the target location beta power had less desynchronization with greater angular disparity.

#### Left and right posterior clusters

##### Alpha band (8–12 Hz)

**Figure 8:**
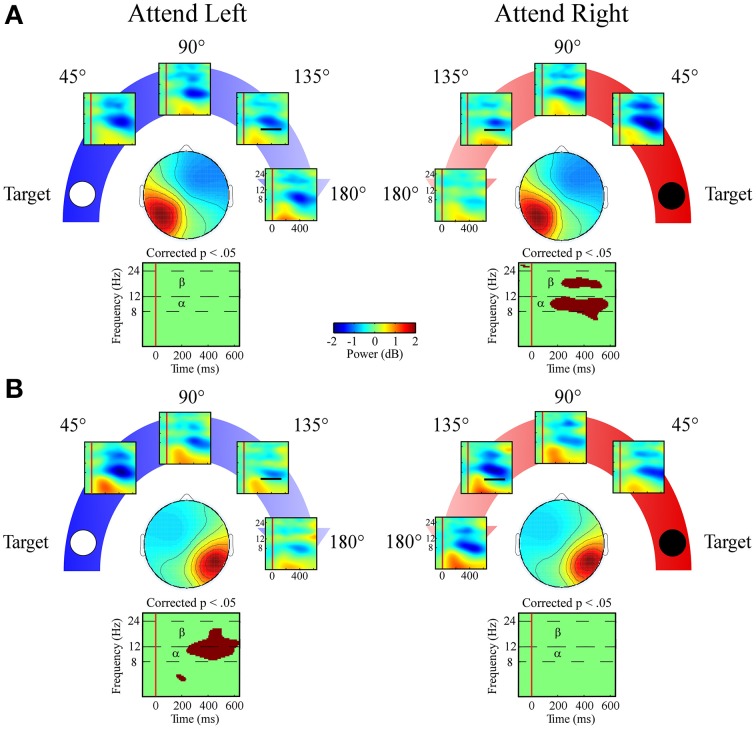
**ERSPs from lateral posterior ICA clusters**. Event-related spectral perturbation plots for the **(A)** Left Posterior cluster and **(B)** Right Posterior cluster shown separately for left and right target locations. Plots below each scalp map highlight time-frequency points for which *p* < 0.05 in a *t*-test across angular disparity after correcting with False Discovery Rate. Mean ERSP power across angular disparity was quantified for alpha (α, 8–12 Hz) and beta (β, 12–24 Hz) bands within a 250–550 ms time window (indicated by black bar in the 135° ERSP plot).

Alpha band measures in both lateral posterior clusters exhibited a target location x angular disparity interaction [left posterior cluster: *F*_(1, 34)_ = 7.9; *p* = 0.008; right posterior cluster: *F*_(1, 34)_ = 22.9; *p* < 0.001; Figures 8A,B]. Only when the target location was contralateral to each cluster's mean scalp map did these clusters have linear decreases in alpha desynchronization as the distractor sound was presented farther from the target location.

##### Beta band (12–24 Hz)

Both lateral posterior clusters exhibited a target location x angular disparity interaction [left posterior cluster: *F*_(1, 34)_ = 7.4; *p* = 0.01; right posterior cluster: *F*_(1, 34)_ = 4.1; *p* = 0.049] within the beta band. As in the alpha band, only when attending to the contralateral target location did the lateral posterior clusters show a linear decrease in beta desynchronization power as the distractor sound was presented farther from the target location (Figures 8A,B).

## Discussion

The objective of this experiment was to define cortical activity associated with the spatial allocation of auditory attention. The strategy was to have participants attend to one spatial location on either the far left (−90°) or far right (+90°) of midline and then to test for linear increases or decreases in evoked responses to distractor sounds as a function of distance from the target location (angular disparity). EEG slopes had two distinct peaks with positive (~300 ms) and then negative (~430 ms) slopes relative to the attended location, which were followed by a positive peak that had a more gradual taper over time (~550–750 ms). The three slope peaks had distinct scalp topographies: the first was maximal at frontocentral sites, the second was focused over parietal areas, and the third was lateralized to frontal sites contralateral to the attended location. Independent component analysis identified six components found in most participants that corresponded in time and topography to the first and second slope peaks. The results from scalp EEG and ICA measures will be discussed individually to explore how they may code for auditory attentional gradients, and will then be related to current theories on cortical spatial attention networks.

### Event-related potential slope measures and attention gradients

Successful orientation of attention was confirmed by accurate performance in responding to targets and the avoidance of false alarms to non-target distractors. The EEG results indicate an attention effect because the same stimuli elicited different EEG responses depending on target location. We note that this study was designed to identify neural activity associated with putative attention gradients, but did not assess how the EEG gradients related to behavior. Additional work is now needed to define the functional significance of the EEG measures, such as by examining variations in target responses or by changing the task to include behavioral measures to targets and distractors.

Previous studies that used auditory ERPs to examine attention gradients employed tasks were the stimuli were presented rapidly over a relatively small range of locations (Teder-Salejarvi and Hillyard, 1999; Teder-Salejarvi et al., 1999; Arnott and Alain, 2002a,b). Perceptual load is high under these conditions, which has been shown to have a strong influence on attentional orienting (Lavie, 2005; Yurgil and Golob, 2013) and is an important consideration when interpreting neural measures associated with attention gradients. Under conditions of high perceptual load the main auditory ERP measures were a negative slow wave from ~100–500 ms and the P3b (or P300) potential which is associated with target processing. Results showed that the amplitudes of the negative slow wave and P3b potentials decreased with increasing distance between the stimulus and target location, with a steeper reduction for the P3b (Teder-Salejarvi and Hillyard, 1999; Teder-Salejarvi et al., 1999; Arnott and Alain, 2002a,b). In the present study perceptual load was rather low; stimuli were presented at a slow rate and there was 45° of separation between stimulus locations. The early onset of attentional effects in these studies (~100 ms) relative to the present finding (~200 ms) may reflect greater perceptual loads inducing earlier sensory filtering (Lavie, 2005). However, in low-load auditory cued attention tasks the influence of attention is manifest at about the same time (~100 ms) as in the above tasks with high perceptual load (Hugdahl and Nordby, 1994; Golob et al., 2002; Bennett et al., 2004). The tri-phasic slope response is a novel finding as well, and under conditions of rapid stimulus presentation (several/sec) would likely not have enough time to develop between stimuli. Taken together, factors such as perceptual load, in particular stimulus presentation rate, as well as the distinction between sustained attention vs. trial by trial cuing task are likely important factors for the specific auditory ERP responses that relate to attention gradients.

The tri-phasic slope response to distractor sounds may represent processing within frontal and parietal regions that mediate attention shifts from the target location to the distractor locations, and then back to the target location. On the basis of a common functional response to distractors, peak latency and scalp topography; the first slope peak at ~300 ms is likely related to the P3a. The P3a is a well-studied evoked response associated with attention capture that is elicited by distractors or novel stimuli in the context of a given task (Friedman et al., 2001; Polich, 2007). The second slope peak occurs about 100 ms later over the parietal cortex and, as discussed below, may represent the top-down control of reorienting back from the distractor location to attended location. The third slope peak begins ~550 ms after distractor onset, is maximal at frontal sites contralateral to the target location, and reflects the sustained frontal slow waves observed in our previous study (Golob and Holmes, 2011). The functional role of the third peak is unclear. Given its specificity in terms of spatial tuning and correspondence to the target location, it is unlikely to reflect slow waves such as the contingent negative variation that develop before an expected stimulus (Brunia, 1999). The first and third peaks are both maximal at frontal sites and have positive slopes, and thus may indicate re-establishment of initial attention gradient. However, there may be important differences between the three peaks because in addition to having contralateral topography the slope of the third peak is also less linear than the first two slope peaks.

Attentional gradients assessed using ICA were manifest in the ERP and ERSP measures. Both the ERP and ERSP measures reflect changes in EEG dynamics relative to a prestimulus baseline. However, ERSPs index a change in oscillatory activity that does not have to be phase locked (i.e., variable phases across trials) while phase-locking is required for ERPs (Makeig, 1993). Therefore, ERSPs represent additional information about neuronal activity that may not be evident in phase-locked ERPs. The six independent components reflected activity that temporally and topographically corresponded to the first and second slope peaks seen in the analysis of scalp channel data. We speculate the reason none of the components appeared to cleanly map onto the third slope peak is that the corresponding slow wave has a widespread topography and thus may be represented by multiple components. This may also reflect high-pass filtering in the ICA analysis.

### Evoked potentials and relations to functional anatomical models of spatial attention

There are two main theories on how spatial attention is represented within a frontoparietal network: the right hemisphere dominance model (Heilman and Van Den Abell, 1980) and the opponent process model (Kinsbourne, 1977, 1993; Reuter-Lorenz et al., 1990). Both were motivated by the symptoms of hemineglect following brain damage, and are mostly based on studies in the visual modality. The right hemisphere dominance theory proposes that right hemisphere regions such as the temporal-parietal junction and inferior prefrontal cortex guide attention in both the left and right visual fields, while homologous regions in the left hemisphere code for just the right hemispace (Heilman and Van Den Abell, 1980; Mesulam, 1981).

In contrast, Kinsbourne's opponent process model posits that regions in each hemisphere mediate attentional bias toward the contralateral hemifield, but these regions also inhibit their cognate in the other hemisphere (Kinsbourne, 1970, 1977, 1987). To a first approximation the right hemisphere dominance and opponent process models are not mutually exclusive. For example, Corbetta and Shulman (2002) propose a two attention network model that possess features of both models described above. One network is a bilateral dorsal attention network which instantiates contralateral spatial coding (much like the activation orienting model), and the other network is a right hemisphere ventral attention network for non-spatial functions such as novelty detection, arousal, and vigilance that can have indirect effects on the dorsal attention network.

Our findings had elements to be expected of both the right hemisphere dominance and opponent process models, but are most consistent with the dorsal and ventral attention framework. There were three frontal components: a central component that showed a gradient regardless of target location, and two lateral components that were active when contralateral to the target location. The finding that a pair of left and right lateralized components were active contralateral to the attended location is expected according to the opponent process model (Kinsbourne, 1987). Although a detailed comparison of curve fitting was not done, Figure 5 suggests that left frontal component processing was tuned based on the hemispace in which non-target sounds were presented. In contrast, the right lateral component had progressive increases in activation as non-targets were presented further from the left target location. This may indicate that, among the two lateral frontal components, the right codes for a more refined attentional representation of space. The frontal gradient responses preceded those of the posterior independent components by ~100 ms, which is consistent with the fact that secondary auditory cortex has dense connections to both inferior parietal and prefrontal cortex (Goldman-Rakic, 1988; Romanski et al., 1999; Petrides and Pandya, 2009). We speculate that this initial gradient, which likely reflects frontal sources, may index a saliency code (Itti and Koch, 2001) for distractors that is derived from comparing distractor locations relative to the target location. Human intracranial recording studies using a non-spatial target detection task show that the P3 is generated in a network of prefrontal and temporal regions (Halgren et al., 1998), and occurs in the same time range as the initial slope peak in the present study.

Parietal measures also indicated gradient-like slopes for distractor processing relative to the target location. Unlike the frontal gradient, wherein for each target location there was symmetry between a central component and a contralateral component, some of the parietal independent component responses were not symmetrical when attending to the left vs. right target location. Although the precise sources of the ERP and independent component measures are not known, they likely reflect, in part, parietal cortex activity that is vital for disengaging and shifting attention in visual tasks (Posner et al., 1984; Colby and Goldberg, 1999; Husain and Nachev, 2007; Shulman et al., 2009). Circumstantial evidence for the parietal gradient having a role in reorienting attention back to the target is provided by observations that ~400–600 ms is when the auditory attentional blink starts to subside (Ward et al., 1996), and inhibition of return is evident (Mondor, 1999). More direct evidence shows that cues to shift attention in a dichotic listening task are associated with activity in the right temporal parietal junction at about the same time period (Larson and Lee, 2014). Lastly, the second slope peak is similar in terms of latency and topography to the reorientation negativity peak, which also follows the P3a peak (Schroger and Wolff, 1998; Berti et al., 2013). The reorientation negativity is considered a marker for reorienting attention back to a primary task after a distracting stimulus (Berti, 2008). It is worth noting, however, that the reorientation negativity can also overlap with the time period of the third slope peak. Future work would be needed to rigorously examine potential relations between the gradient profiles in the present study and the reorientation negativity.

Among the three parietal ICA components the central posterior cluster showed a negative slope centered around 400 ms when attending to the left or right target location, indicative of a bilateral coding of attention allocation within auditory space. This profile is similar to the frontal central component but with an opposite slope. The right parietal cluster was particularly interesting given the debate about right hemisphere dominance as it had both a positive slope ~400 ms when attending to the ipsilateral right target location, and a negative slope ~500 ms when attending to the contralateral left target location. Therefore, when auditory attention is directed to the right side of space the ICA ERPs from both the central posterior and right posterior clusters have spatial gradient around the same time period, but with slopes in opposite directions. Perhaps the right posterior cluster is showing the opposite gradient compared to the central posterior cluster as a means of representing the coding for ipsilateral instead of contralateral attention. Taken together, the right posterior ICA cluster may show that mechanisms where the right parietal cortex directs attention to either hemifield at slightly different times, and comports well with the right dominance and ventral attention system models (Heilman and Van Den Abell, 1980; Mesulam, 1981; Corbetta et al., 2008). The ERPs from the left posterior cluster were not modulated by spatial attention, and will be discussed later.

### Neural oscillations and attention gradients

Oscillatory power within the alpha and beta frequency bands decreased starting around 250 ms after distractor onset in both frontal and posterior ICA clusters. However, only the event-related desynchronization within posterior ICA clusters displayed an effect of angular disparity. Event-related desynchronizations reflect consistent reductions in EEG power across individual trials, but unlike ERPs the phase of the evoked EEG relative to stimulus onset can be variable across trials. Event-related desynchronizations have been related to increased cortical activity during information processing (Pfurtscheller, 1992; Pfurtscheller and Lopes da Silva, 1999; Makeig et al., 2004). The central posterior ICA cluster showed a gradient pattern within the beta band when attention was directed to either the left or right hemispace, which matches that cluster's ERP result. The alpha frequency band had more specificity, as a gradient was only present when attention was directed toward the right hemispace. Thus, rightward-directed attention appears to be more broadly coded by spanning the alpha and beta frequency bands within the central posterior cluster. Other studies have found that event-related power changes in alpha and beta bands can also index attention (Rihs et al., 2009; Tan et al., 2013).

Both the left and right lateral posterior components showed an auditory spatial attention gradient when attention was directed to the contralateral hemispace in both the alpha and beta frequency bands. This differs from the lateral ICA ERP measures, where only the right posterior cluster displayed an auditory spatial attention gradient to both ipsilateral and contralateral directed attention. The current findings examined EEG power within components, and additional study would be needed to examine associations between components. Overall, the posterior components support the opponent process model when viewed using EEG oscillation measures while evoked responses were more consistent with the right hemisphere dominance model.

### Attention-related vs. head-related reference frames

The scalp EEG data and most of the independent component clusters (5 out of 6 clusters) showed differences across conditions that coded for space relative to the attended location rather than the spatial location with respect to a head-centered reference frame. Thus, the current study shows the majority of the measured cortical potentials reflect a transformation of sound location into a spatial attention-based coordinate system. Only one early ERP component (120–200 ms) from the left parietal cluster was tuned to absolute space, with a progressively smaller evoked response as sounds were presented farther to the right side of space. Evidence from single-unit and field potential recordings suggests the auditory modality uses a population code in which two distinct populations of neurons distributed in both auditory cortical hemispheres code for locations within one hemispace (Harper and McAlpine, 2004; Salminen et al., 2012), with potentially a third population centered at midline (Kitzes et al., 1980). Based on these studies, one would expect to find an EEG signal coding for absolute space in each hemisphere or not to find one at all due to lack of spatiotemporal coherence in neural signals. A right-lateralized signal coding for absolute space may have been dominated by activity from right-lateralized structures specifically active when representing space relative to a target location. Notably, the coding of absolute space was ~150 ms earlier than the first peak of the attention-related spatial gradient, which would be anticipated because the processing of absolute sound location must occur before an attention-based reference frame can be established.

## Conclusion

This study used EEG to identify a tri-phasic response that reflects spatial relations between an attended location and distractor locations. The progressive changes in various neural measures as a function of distance from the attended location may indicate neural coding of auditory spatial attention gradients. The results were broadly consistent with the dorsal and ventral system framework, and exhibited aspects of opponent processing and hemispheric dominance theories of spatial attention. Taken together, the findings show a rich coding of space that reflects the temporal interplay of frontal and parietal regions, with neural signaling that likely reflects rapid shifts of attention from a target to a distractor, and back to the target location.

### Conflict of interest statement

The authors declare that the research was conducted in the absence of any commercial or financial relationships that could be construed as a potential conflict of interest.
